# Resting-state brain functional connectivity in patients with chronic pain who responded to subanesthetic-dose ketamine

**DOI:** 10.1038/s41598-019-49360-1

**Published:** 2019-09-09

**Authors:** Yasushi Motoyama, Yoshitetsu Oshiro, Yumiko Takao, Hitoaki Sato, Norihiko Obata, Shinichiro Izuta, Satoshi Mizobuchi, Shigeyuki Kan

**Affiliations:** 1 Department of Surgery Related, Division of Anesthesiology, Kobe University Graduate School of Medicine, Kobe, Japan; 2 Department of Anesthesiology and Pain Medicine, Hyogo College of Medicine, Nishinomiya, Hyogo Japan; 3 Department of Anesthesiology and Intensive Care Medicine, Osaka University Graduate School of Medicine, Osaka, Japan

**Keywords:** Predictive markers, Brain imaging

## Abstract

Ketamine has been used to treat chronic pain; however, it is still unknown as to what types of chronic pain is ketamine effective against. To identify the effect of administration of subanesthetic-dose ketamine in patients with chronic pain and to clarify the mechanism of the effect, we retrospectively investigated brain functional connectivity using resting-state functional magnetic resonance imaging (rs-fMRI). Patients were divided into responders (Group R: ≥50% improvement on Numerical Rating Scale) and non-responders (Group NR). We compared the differences in terms of brain functional connectivity by seed-to-voxel correlation analysis. Two-sample *t*-test revealed significant lower connectivity between the medial prefrontal cortex (mPFC) and precuneus in Group R. We also found a significant negative correlation between the improvement rate and functional connectivity strength between the mPFC and precuneus. These findings suggest that subanesthetic-dose ketamine is effective in patients with chronic pain whose brain functional connectivity between the mPFC and precuneus is low. We believe that the current study explored for the first time the correlation between brain functional connectivity and the effect of subanesthetic-dose ketamine for chronic pain and indicated the possibility of use of the predictive marker in pharmacological treatment of chronic pain.

## Introduction

In recent years, the rapid and sustained antidepressant effect of subanesthetic-dose ketamine has received considerable attention^1–3^. In these cases, brain imaging studies^4,5^ using functional MRI (fMRI) to evaluate blood flow dynamic responses related to human and animal central nervous system activities were conducted. Changes in central nervous system activity following ketamine administration have been observed in healthy adults and patients with depression. However, to the best of our knowledge, no previous study has examined the relationship between brain activity in patients with chronic pain and their responses to subanesthetic-dose ketamine.

Ketamine is a general anesthetic used as an N-methyl-d-aspartate receptor (NMDA) antagonist since the 1960s^6–8^. In a study involving a neuropathic pain animal model, it was reported that the spared nerve injury model of neuropathic pain induced depressive behavior in rats and a single subanesthetic dose of ketamine successfully treated this depressive behavior^9^. Ketamine has been used to treat chronic pain; however, it is still not known what kind of chronic pain ketamine is effective against. Identification of patients who respond to ketamine by noninvasive examination can provide substantial clinical benefits to treatment of patients with chronic pain.

fMRI is a noninvasive examination for brain activity. Resting-state fMRI (rs-fMRI) is a research method for targeting baseline brain activity at rest. The rs-fMRI signal displays spontaneous fluctuations associated with the temporal patterns of neural activity. The correlation of these spontaneous fluctuations in distant brain regions is called brain functional connectivity, and it is believed to be the basis of communication within the brain network. Brain functional connectivity is used in many different clinical applications^10^; it is also used in the field of pain research, and many studies have been conducted on subjects with chronic pain^11^.

In this study, to identify the effect of administration of subanesthetic-dose ketamine to patients with chronic pain and to clarify the mechanism of the effect, we examined the differences between patients who responded to the treatment and those who did not based on brain functional connectivity using fMRI. By assessing rs-fMRI images prior to the treatment, we managed to identify the key region of therapeutic response to subanesthetic-dose ketamine in patients with chronic pain.

## Materials and Methods

### Participants

**Figure 1:**
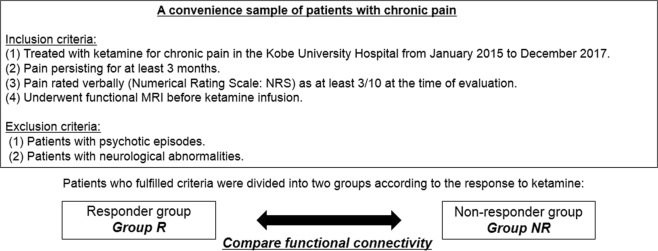
Overall study design. Responder was defined as an individual with ≥50% reduction as per the Numerical Rating Scale score (NRS). NRS (Numerical Rating Scale): 0 = “no pain” and 10 = “the worst pain imaginable”.

A convenience sample of patients with chronic pain was included in this study (Fig. 1). The criteria for inclusion in this study were as follows: (1) treated with subanesthetic-dose ketamine for chronic pain in the Kobe University Hospital from January 2015 to December 2017; (2) pain persisting for at least 3 months; (3) pain rated verbally (Numerical Rating Scale (NRS): 0 = “no pain” and 10 = “the worst pain imaginable”) as at least 3/10 at the time of evaluation; and (4) underwent fMRI prior to ketamine infusion. We excluded patients with previous psychotic episodes and/or neurologically abnormal MRI results. The study protocol was approved by the Institutional Review Board of Kobe University Hospital, and the study was conducted in accordance with the Declaration of Helsinki. Prior to performing fMRI, a written informed consent was obtained from each patient.

### Ketamine treatment and experimental design

Eligible patients completed structural MRI and rs-fMRI following medical assessment. Within 30 min after MRI imaging, ketamine (0.3 mg/kg) was administered intravenously to all patients for 30 min. Ketamine treatment is usual care at our facility for the types of chronic pain that is resistance to treatment such as nerve block and medication. Three physicians who examined the patient at outpatient department decided to treat patient with subanesthetic-dose ketamine. The dosage and administration method of ketamine were based on the conventionally method in our facility. The patients were then asked to complete pain ratings, including NRS, Hospital Anxiety and Depression Scale (HADS^12^), and Pain Catastrophizing Scale (PCS^13^) prior to ketamine administration and 60 min following the end of ketamine infusion. Further, they were divided into two groups according to their pain response: responders (Group R; defined as ≥50% reduction as per the NRS score) and non-responders (Group NR)^14^.

### rs-fMRI acquisition

Neuroimaging was performed on a 3-Tesla Siemens Skyra scanner (https://www.healthcare.siemens.com/magnetic-resonance-imaging/3t-mri-scanner/magnetom-skyra) with a 20-channel head coil. Prior to performing rs-fMRI, the patients were given the following instructions: “Close your eyes, relax, and do not think of anything in particular. Do not fall asleep.” Scans were then acquired using single-shot gradient-recalled echoplanar imaging. The acquisition parameters were set as follows: TR: 2000 ms, TE: 30 ms, flip angle: 90°, voxel size: 3.75 × 3.75 × 3.0 mm^3^ with 0.6 mm gap, field of view: 240 × 240 mm, matrix size: 64 × 64, number of slices: 40, number of scans: 300, and scan time: 10 min. High-resolution 1-mm isotropic T1-weighted images of the entire brain were also collected to provide anatomical information to superimpose functional activation maps.

### fMRI data analysis

Preprocessing procedures were performed with the CONN functional connectivity toolbox (ver.17.c;www.nitrc.org/projects/conn) and SPM12 (www.fil.ion.ucl.ac.uk/spm/). Briefly, images of each patient were first realigned (motion corrected) and corrected for slice timing. Structural images were normalized to a Montreal Neurological Institute’s echoplanar imaging template with affine registration, followed by nonlinear transformation. Then, the normalization parameters determined for the structural volume were applied to the corresponding functional image volumes for each subject. Finally, the images were smoothed with a Gaussian kernel of 8 mm at full width at half maximum. Before averaging of individual voxel data, the denoising processes were performed. CONN implements the component-based noise correction method (CompCor) strategy for physiological and other noise source reduction, additional removal of movement, and temporal covariates, temporal filtering and windowing of the residual blood oxygen level-dependent (BOLD) contrast signal^15^. One of the denoising processes is the removal of the effect of low-frequency drift and high-frequency noise by a band pass filter (0.008–0.09 Hz). The other is removal of non-neural activity effect from the ventricular regions, the white matter, and head motion by multiple regression. Individual correlation maps were generated in the CONN by extracting the mean BOLD time course from each region of interest (ROI) and calculating correlation coefficients with the BOLD time course of each voxel throughout the whole brain. The resulting coefficients were converted to normally distributed scores using Fisher’s transformation to give maps of voxel-wise functional connectivity for each ROI for each subject. The value of each voxel throughout the whole brain represents the relative degree of functional connectivity with each seed. These maps were subsequently used for second-level analysis of relative functional connectivity using a two-sided independent t test, implemented in the CONN, to investigate differences in seed-to-voxel connectivity between groups.

Seed-to-voxel correlation analysis was performed to investigate the difference in brain functional connectivity between Group R and Group NR. The seeds used for analysis were the medial prefrontal cortex (mPFC), posterior cingulate cortex (PCC), anterior cingulate cortex, and anterior insular cortex as the specific regions of the default mode network (mPFC–PCC) and the salience network (anterior cingulate cortex–anterior insular cortex) because there is a link between these networks and the chronicity of pain^16,17^. It has been also reported in previous studies that ketamine modulates Default Mode Network (DMN) and that resting state network such as DMN is involved in its treatment effect^18^. A previous study reported that chronic pain alters brain functional connectivity from nociceptive circuits to emotional circuits^19^, in addition, glutamatergic NMDA receptors are abundant in the subcortical region^20^; thus, we also defined the seed in the amygdala and nucleus accumbens. These seeds are provided in the CONN. Subcortical seeds, the amygdala and nucleus accumbens, are from FSL Harvard-Oxford Atlas maximum likelihood subcortical atlas (HarvardOxford-sub-maxprob-thr25-1mm.nii). Seeds as the network defined from CONN’s Independent Component analyses of healthy control dataset (497 subjects). These seeds were 10 mm diameter spheres. The spatial coordinate of these network seeds were presented here; mPFC (x, y, z; 1, 55, −3), PCC (x, y, z; 1, −61, 38), anterior cingulate cortex (x, y, z; 0, 22, 35), left anterior insula cortex (x, y, z; −44, 13, 1), right anterior insula cortex(x, y, z; 47, 14, 0). The reasoning for identification and use of these seeds is described in greater detail by the originators of CONN^15^. We performed voxel-wise statistical analysis over the whole brain using an uncorrected level (p < 0.001) before a familywise error rate (FWE) correction was applied at the cluster level (p < 0.05)^21^.

Based on the results of the seed-to-voxel correlation analysis described below, we also performed independent component analysis (ICA). ICA has been widely used to identify brain networks in resting-state and task-based fMRI data. The fMRI data were submitted to a subject-wise group ICA implemented in the CONN. The group ICA was performed with 20 factors and a dimensionality reduction of 64 as a default setting of CONN.

### Statistical analysis

We used paired *t*-test to examine the effect of ketamine treatment on pain severity and other scores between pre- and post-ketamine infusion. All clinical data were also compared using two-sample *t*-test between Group R and Group NR.

To examine changes in brain functional connectivity between the groups, a random effect, two-sample *t*-test was performed. The statistical results were used to determine the brain regions showing significant differences in terms of correlation to each region defined as the seed. The average of the Z-values from all the voxels in the correlated regions as the functional connectivity strength and NRS reduction rate between pre- and post-ketamine infusion were analyzed using Spearman’s rank correlation test. All statistical analyses were performed using MATLAB R2017b. (https://jp.mathworks.com/products/matlab.html) p < 0.05 was considered statistically significant.

## Results

### Patient data and pre- and post-ketamine infusion clinical score

Between January 2015 and December 2017, 33,286 patients visited our outpatient clinic. A total of 30 patients agreed to undergo pre-treatment fMRI, and 24 patients met the inclusion criteria. Patient details are summarized in Table 1 (age range, 27–81 years; mean = 56 ± 3.5 years; Male/female: 19/5).

**Table 1:** Subject data.

	Subject No.	Age	Sex	Diagnosis	Dosage of ketamine (mg/kg)	Medication	Clinical Scores Pre - Post Ketamine			
							NRS	HADS-D	HADS-A	PCS
Group R	1	52	M	TCS	0.299	Tra, Gab	4 - 1	15 - 12	4 - 0	22 - 20
	2	45	F	FPD	0.300	Pgb, Dul	9 - 1	6 - 1	5 - 0	33 - 19
	3	79	M	PHN	0.301	Pgb, Ami	5 - 0	10 - 1	9 - 3	19 - 14
	4	31	M	TCS	0.295	Pgb, Dul	9 - 2	18 - 18	17 - 9	48 - 36
	5	50	M	LSS	0.303	Dul	3 - 1	12 - 11	12 - 11	48 - 42
	6	74	M	PHN	0.294	Pgb, Dul	5 - 2	7 - 7	1 - 1	36 - 30
	7	81	F	PHN	0.303	Tra	6 - 0	7 - 3	1 - 2	42 - 34
	8	66	M	LSS	0.309	—	5 - 2	0 - 3	0 - 7	36 - 38
	9	68	M	PTPS	0.308	Tra	4 - 2	13 - 11	12 - 13	44 - 44
	10	77	M	PHN	0.300	Pgb, Dul	4 - 0	6 - 3	0 - 2	26 - 15
	11	27	M	FPD	0.298	Pgb	7 - 3	10 - 9	5 - 4	28 - 27
	12	49	F	FPD	0.308	Pgb, Dul	5 - 0	4 - 0	7 - 1	16 - 3
Group NR	13	50	M	TCS	0.298	Tra, Gab, Dul	5 - 4	1 - 0	6 - 0	30 - 26
	14	66	M	FPD	0.308	Tra	3 - 2	6 - 1	5 - 0	36 - 18
	15	58	F	FBSS	0.306	Tra, Pgb	5 - 3	6 - 1	9 - 9	29 - 35
	16	23	F	TCS	0.299	—	7 - 5	13 - 10	11 - 4	37 - 33
	17	70	M	PHN	0.308	Pgb, Dul	4 - 4	1 - 2	3 - 5	23 - 25
	18	68	M	LSS	0.300	Tra	4 - 3	6 - 0	5 - 0	12 - 6
	19	43	M	TCS	0.294	Tra, Pgb	3 - 2	1 - 1	3 - 1	21 - 15
	20	57	M	FPD	0.299	—	9 - 6	7 - 4	7 - 0	50 - 44
	21	29	M	FBSS	0.299	Dul	4 - 3	6 - 12	2 - 10	26 - 40
	22	46	M	CS	0.300	Dul	3 - 3	8 - 7	7 - 8	35 - 34
	23	78	M	LSS	0.294	Pgb, Dul	10-/6	9 - 2	14 - 11	47 - 46
	24	53	M	CRPS	0.307	—	3 - 3	7 - 6	9 - 3	24 - 21
	Mean						5.3 - 2.4	7.5 - 5.2	6.4 - 4.5	32 - 28
							*p* < 0.05	*p* < 0.05	*p* < 0.05	*p* < 0.05

Paired *t*-test showed a significant difference between pre-post-ketamine scores on NRS, HADS-A, HADS-D, and PCS (*p* < 0.05). Twelve (three females) patients showed an improvement in NRS score of ≥50% following ketamine treatment and were classified into Group R. The remaining 12 (two females) were classified into Group NR.

Group R: Responders, Group NR: Non-responders.

TCS, traumatic cervical syndrome; FPD, functional pain disorders; PHN, post-herpetic neuralgia; LSS, lumbar spinal canal stenosis; PTPS, post-thoracotomy pain syndrome; FBSS, failed back surgery syndrome; CS, cervical spondylosis.

Tra, tramadol; Gab, gabapentin; Pgb, pregabalin; Dul, duloxetine; Ami, amitriptyline

NRS (Numerical Rating Scale): 0 = “no pain” and 10 = “the worst pain imaginable”. HADS-A (Hospital Anxiety and Depression Scale-Anxiety). HADS-D (Hospital Anxiety and Depression Scale-Depression). PCS (Pain Catastrophizing Scale).

Paired *t*-test revealed a significant effect of treatment on NRS, HADS-A, HADS-D, and PCS. Twelve (three females) patients showed an improvement on NRS of ≥50% following ketamine treatment and were classified into Group R, and the remaining 12 (two females) were classified into Group NR. There was no significant difference in terms of age, dosage of ketamine, pre-treatment NRS, HADS, and PCS between the two groups (Table 2).

**Table 2:** Summary of subject data and clinical scores of Group R and Group NR prior to the treatment.

	n	Age (years)	Dosage of ketamine (mg/kg)	Pre ketamine clinical score (mean ± SEM)			
				NRS	HADS-D	HADS-A	PCS
Group R	12 (3 female)	58 ± 5.3	0.301 ± 0.0014	5.5 ± 0.5	9 ± 1.4	6.0 ± 1.5	33 ± 3.1
Group NR	12 (2 female)	53 ± 4.7	0.300 ± 0.0014	5 ± 0.6	5.9 ± 1.0	6.7 ± 1.0	31 ± 3.1
		*p* = 0.50	*p* = 0.77	*p* = 0.58	*p* = 0.09	*p* = 0.70	*p* = 0.60

There was no significant difference in terms of age, dosage of ketamine, pre-treatment NRS, HADS, and PCS between Group R and Group NR.

Group R: responders, Group NR: non-responders. NRS (Numerical Rating Scale): 0 = “no pain” and 10 = “the worst pain imaginable”. HADS-A (Hospital Anxiety and Depression Scale-Anxiety). HADS-D (Hospital Anxiety and Depression Scale-Depression). PCS (Pain Catastrophizing Scale).

### Head motion during fMRI Scans

We compared both study groups as for potential head motion. There are some studies that head motion during MRI acquisition affects the analysis result of brain functional connectivity^22–24^. Brain functional connectivity analysis results may be affected if one of the two groups had moved significantly more than the other^25,26^. To evaluate this possibility, we calculated the mean volume-to-volume difference of each of the six rigid body transformation parameters (x-, y-, and z-translation, pitch, roll, and yaw) and compared these values between the two groups by two sample t-test. We found no significant differences in any parameter.

### Functional connectivity analysis between Group R and Group NR

**Figure 2:**
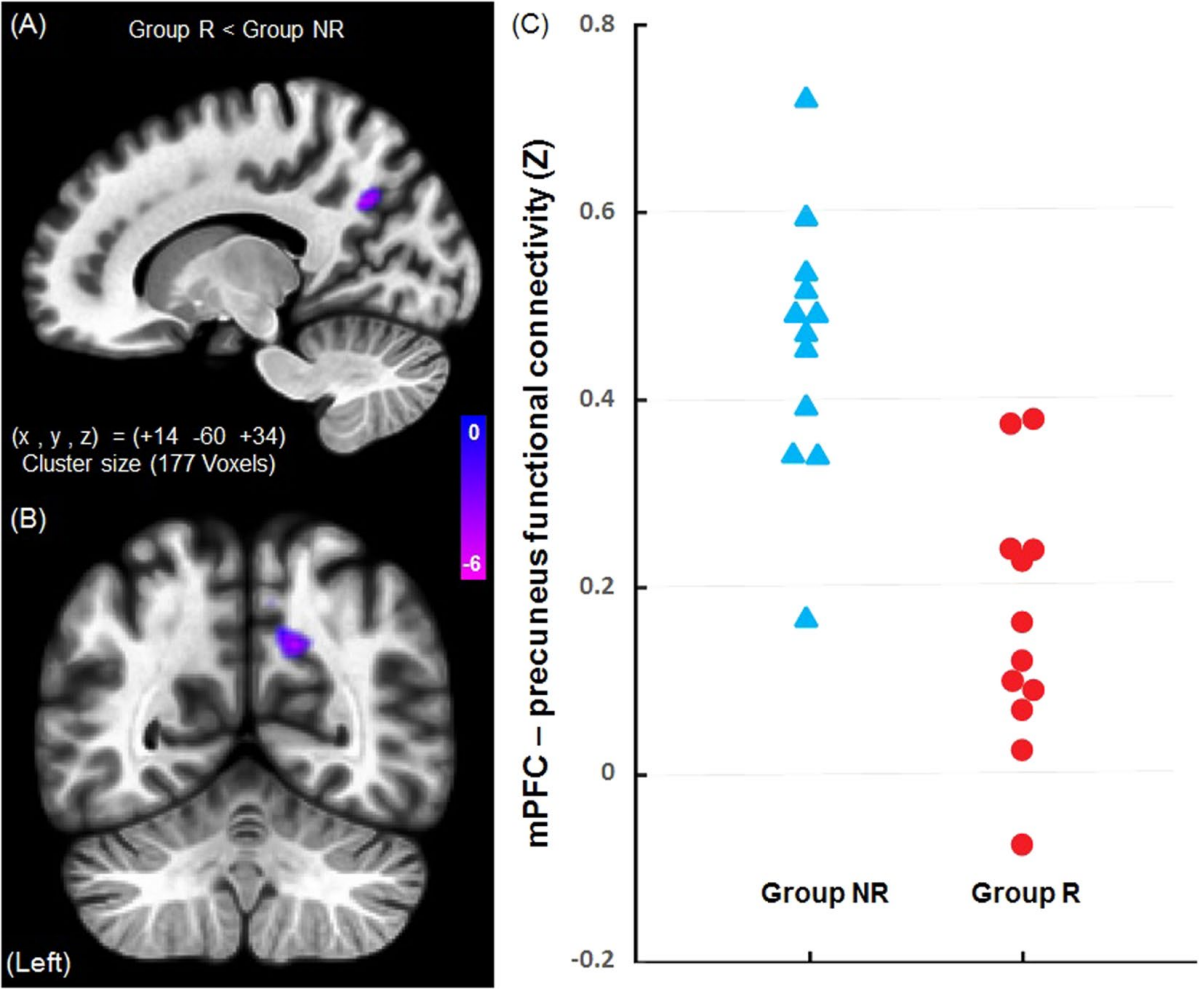
Functional connectivity differences between Group R and Group NR. (**A**,**B**) Group R showed significantly weaker brain functional connectivity between the medial prefrontal cortex and precuneus than Group NR. (voxel level p < 0.001, uncorrected, t < −3.50; cluster level p < 0.05, FWE corrected, number of voxels ≥177). Effects that have not reached the threshold were not displayed. Color scale: t-value. (**C**) The average of the Z-values of each patient extracted as the functional connectivity strength between MPFC and precuneus are shown. That was significantly difference between Group R and Group NR (mean, Group R; 0.1628, Group NR; 0.4589, *p* < 0.05). Group R: responders, Group NR: non-responders.

The result of functional connectivity analysis between Group R and Group NR is shown in Fig. 2. Seed-to-voxel correlation analysis revealed that brain functional connectivity was significantly lower between MPFC and precuneus in Group R (Fig. 2A,B, voxel level p < 0.001, uncorrected; cluster level p < 0.05, FWE corrected). Because of MPFC is an area that is vulnerable to aging and the sample included some elderly subjects, we performed additional analysis. Seed-to-voxel correlation analysis was performed by adjusting the influence of aging with covariate of age in the second level analysis, and almost the same results were obtained as when the influence of age was not adjusted (voxel level p < 0.002, uncorrected; cluster level p < 0.05, FWE corrected). Seed-to-voxel correlation analysis of salience network, nucleus accumbens, and amygdala showed no significant difference between Group R and Group NR.

### Correlation of the mPFC–precuneus functional connectivity and response to ketamine therapy

**Figure 3:**
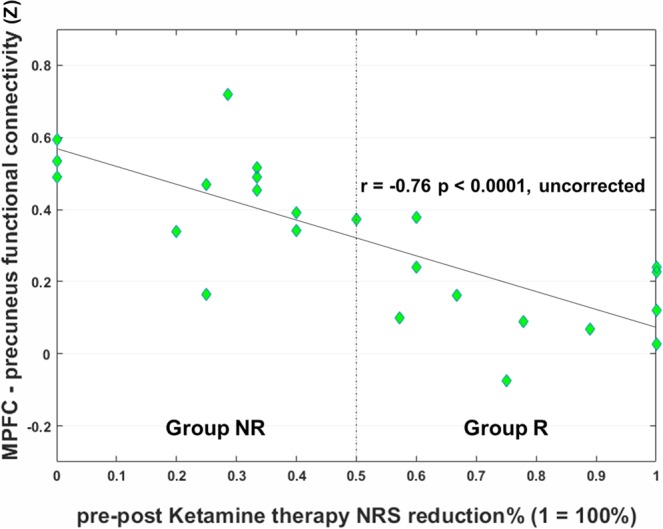
Correlation of the mPFC–precuneus functional connectivity and response to ketamine therapy. Correlation analysis of medial prefrontal cortex–precuneus functional connectivity and pre–post ketamine therapy Numerical Rating Scale (NRS) reduction. There was a significant negative correlation between NRS reduction rate and functional connectivity strength. Group R: responders, Group NR: non-responders. NRS (Numerical Rating Scale): 0 = “no pain” and 10 = “the worst pain imaginable”.

The average of the Z-values of each patient extracted as the functional connectivity strength between MPFC and precuneus are shown in Fig. 2C. The correlation analysis of the Z-values with NRS change revealed a significant negative correlation between the MPFC–precuneus functional connectivity and pre–post ketamine therapy NRS reduction rate (Fig. 3, r = −0.76, *p* < 0.001, uncorrected).

### Independent component analysis

**Figure 4:**
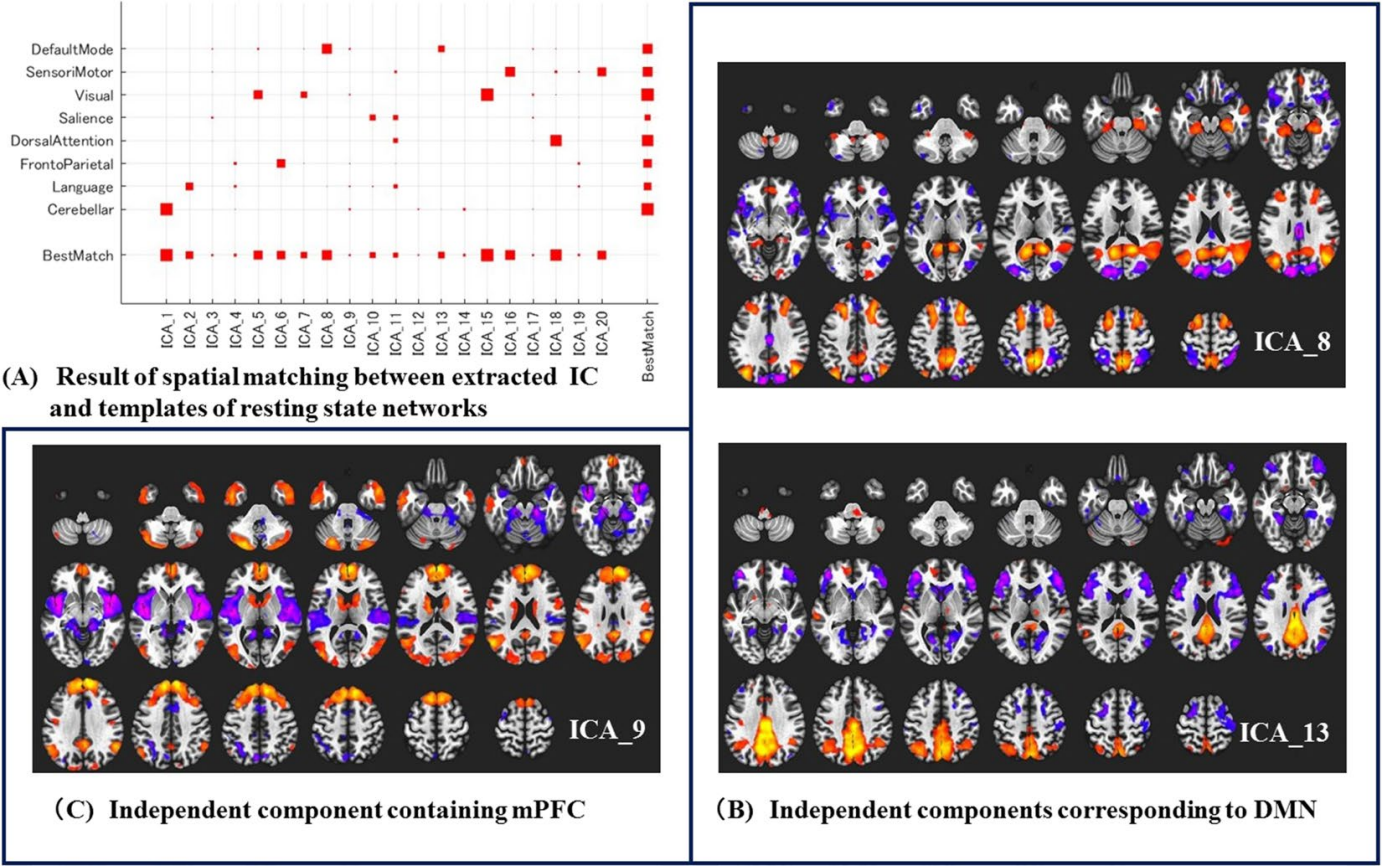
Result of Independent Component Analysis. (**A**) ICA_8 & 13 corresponded to DMN according to spatial matching between extracted IC by group ICA and templates of resting state networks implemented in CONN. The larger the size of the red square, it indicates that the IC matched the template of the specific networks more. (**B**) ICA_8 & 13 mainly consisted of the posterior part of DMN (posterior cingulate cortex/precuneus and inferior parietal lobule). (**C**) ICA_9 contained most part of mPFC (anterior part of DMN). This IC did not contain most part of the posterior cingulate cortex/precuneus. ICA: independent component analysis, IC: independent component, DMN: default mode network, mPFC: medial prefrontal cortex. hot color: brain regions with positive functional connectivity, cold color: brain regions with negative functional connectivity.

Seed-to-voxel correlation analysis indicated that functional connectivity between mPFC and precuneus was associated with therapeutic effect of subanesthetic-dose ketamine. Both mPFC and precuneus are the core brain regions of DMN^27^. To consider the possibility that our result relates to alterations of DMN, we performed group ICA. We identified 20 independent components within the fMRI data, and we selected two independent components (ICs) corresponding to DMN according to spatial matching between extracted independent components by group ICA and templates of resting state networks implemented in CONN (Fig. 4A,B). However, these ICs did not contain most part of the mPFC used in the seed-to-voxel correlation analysis, so we selected another IC containing mPFC (Fig. 4C). We performed two-sample t-test with these ICs, but there was no significant difference between Group R and Group NR.

## Discussion

In this study, we revealed that the brain functional connectivity of the mPFC and precuneus was significantly lower in patients with chronic pain who responded to subanesthetic-dose ketamine. In addition, we found that there was a significant negative correlation between the improvement rate of pain following ketamine treatment and the functional connectivity strength between the MPFC and precuneus. To the best of our knowledge, no study has examined the relationship between brain activity in patients with chronic pain and responses to subanesthetic-dose ketamine to date. The study findings demonstrated that ketamine may be effective in treating patients with chronic pain whose functional connectivity between the mPFC and precuneus is low. Our study could be an early finding in demonstrating the effects of administration of subanesthetic-dose ketamine on patients with chronic pain.

In recent years, ketamine has been used as a remarkable research target and a promising drug for multiple psychiatric disorders. Several neuroimaging techniques have been used to examine the effects of ketamine at the local and whole-brain levels. Among these techniques, fMRI is characterized by higher spatial and temporal resolution without risk of radiation exposure and has proven invaluable to the studies of ketamine^5^. Among brain imaging studies, some report that on ketamine administration to healthy adults, BOLD responses were observed. Sprenger reported that ketamine reduced activation of the thalamus, insula, secondary somatosensory cortex, cingulate cortex, and prefrontal cortex in healthy adults when thermal stimulation was applied^28^. Niesters reported that low doses of ketamine activated the anterior cingulate cortex, orbital frontal cortex, insula, and brain stem in healthy adults, and they concluded that ketamine reactivates the descending pain suppression system^29^. Li reported that subanesthetic dose ketamine decreased brain functional connectivity between PCC and mPFC and this functional connectivity decrease correlated with glutamatergic changes in perigenual anterior cingulate cortex^18^. These studies on ketamine included ketamine administration to healthy volunteers, and we found no study of brain imaging that examined the effect of administration of subanesthetic-dose ketamine on patients with chronic pain. Although the dose of ketamine was not subanesthetic-dose recommended by the ketamine consensus guidelines^30,31^, there was one case report and one study of a patients with chronic pain who was treated with ketamine and the BOLD changes were observed. Becerra reported that ketamine coma therapy recovered the altered brain functional network of patients with chronic pain, and the recovered state had paralleled as default networks of healthy volunteers^32^. Rachael reported that patients with refractory neuropathic pain who was treated with ketamine (0.5 to 2 mg/kg/h; mean dose 1.1 mg/kg/h) for 6 hour/day for 5 consecutive days^33^. They reported that approximately 50% of patients had a reduction in NRS of 30% or greater at 1 month after intravenous ketamine infusion, and the relationship between temporal summation of pain and pain relief is mediated by default mode network–descending antinociceptive pathway dynamic functional connectivity. In our study, we revealed that in patients with chronic pain who responded to subanesthetic-dose ketamine, the brain functional connectivity between the mPFC and precuneus was significantly lower on rs-fMRI images, suggesting that subanesthetic-dose ketamine is effective in patients with chronic pain whose brain functional connectivity between the mPFC and precuneus is low.

Regarding the mPFC and precuneus, wherein the change in connectivity was observed in our study, the mPFC is considered to be a brain region related to human decision-making process and the descending pain suppression pathway^34^. On the other hand, the precuneus is important for supporting complex cognition and behavior^35,36^, but the complete function of the precuneus is still unknown. Kucyi reported that the brain functional connectivity between the mPFC and PCC/precuneus was enhanced in patients with chronic pain and reported a positive correlation of the mPFC and PCC/precuneus brain functional connectivity with pain rumination^37^. Rikandi reported that the functional connectivity between the mPFC and PCC/precuneus increased in psychotic disorders^38^. The mPFC and precuneus are cortical midline structures (CMS) related to processing self-related information^39,40^. The CMS are regions that respond not only to physical pain stimuli but also to emotional pain stimuli^41^. Meerwijk *et al*. reported in their review of psychological pain that regions of the CMS play important roles in the recognition of psychological pain^42^. In our study, the HADS-D score tended to be higher in Group R than in Group NR, suggesting that ketamine is more effective for pain related to emotion or psychology.

Both mPFC and precuneus are the core brain regions of DMN^27^, and many studies have reported that DMN alters in various chronic pain patients^16,43,44^. To consider the possibility that our result relates to alterations of DMN, we performed group ICA, but we were not able to find any group differences. Because this result is probably due to ICs mainly consisted of the posterior part of DMN (PCC/precuneus and inferior parietal lobule) and did not contain most part of the mPFC, we also compared an additional IC containing mPFC between the two groups. Nevertheless, group differences were not significant. Considering the discrepancy between results of seed-to-voxel correlation analysis and that of group ICA, lower functional connectivity between mPFC and precuneus should be explained according to specific functions of these two regions rather than the alteration of DMN at this time. However, the sample size of this study was the minimum to perform group comparisons, regarding the statistical power. Therefore, further studies are needed to fully understand the relationship between the therapeutic effect of ketamine and the changes of resting-state networks, in particular, DMN.

The present study has a number of limitations. First, this study was a single-center retrospective study, so many potential confounders are not well controlled, and for example, the use of medication may change the connectivity patterns. Second, the number of patients was small, so further examination of larger patient populations is necessary to verify our findings. Third, the pain site and diseases were not unified, and this confounding factor is not ruled out in this study. In future studies, it may be possible to predict the effect of ketamine on individual diseases and pain site by unifying the subjects. Fourth, all subject received ketamine, but there was no placebo control in this study. This result may be a brain functional connectivity change in association with placebo in patients with chronic pain. Based on the present results, it is necessary to carry out a follow-up study using a placebo control group in the future. Lastly, we divided the patients based on a short time pain response to ketamine. The long-term effects of ketamine treatment are recognized in psychiatry, and some patients with chronic pain respond to ketamine in the long term. If we can reveal the marker that can predict long-term pain response to ketamine, it will be a notable contribution to chronic pain therapy.

In this study, we revealed that the brain functional connectivity of the mPFC and precuneus was significantly lower in patients with chronic pain who responded to subanesthetic-dose ketamine. In addition, we found that there was a significant negative correlation between the improvement rate for pain by ketamine administration and the functional connectivity strength between the mPFC and precuneus. Ketamine may be effective in patients with chronic pain whose brain functional connectivity between the mPFC and precuneus is low. The current study is the first to explore the correlation between brain functional connectivity and the effect of subanesthetic-dose ketamine for chronic pain, and it indicates the possibility of a biomarker for the pharmacological treatment of chronic pain.

## Data Availability

The datasets generated during the current study are available from the corresponding author on reasonable request.
